# ASSOCIATION AMONG HISTOLOGICAL FINDINGS SUGGESTIVE OF PAPILLOMA VIRUS ON HEMORRHOIDECTOMY SPECIMENS

**DOI:** 10.1590/S0102-6720201500040009

**Published:** 2015

**Authors:** Soraya Souto da SILVA, Gerson Suguiyama NAKAJIMA, Ricardo Alexandre GUIMARÃES, Flávia da Costa MOURÃO

**Affiliations:** Laboratory of Pathological Anatomy, Department of Pathology and Legal Medicine, School of Medicine, and Getúlio Vargas University Hospital, Federal University of Amazonas, Manaus, AM, Brazil

**Keywords:** Human papillomavirus, Hemorrhoids, Condylomata acuminada

## Abstract

**Background:**

Many researchers studied human Papillomavirus infection in the anal area supposing it represents a risk factor for precursor lesions of anal cancer.

**Aim:**

To study the association between histological findings suggestive of injury by the virus in hemorrhoidectomy specimens.

**Method:**

Prevalence study was carried out based on histopathological analysis of hemorrhoidectomy specimens to find viral cytopathic effects. These findings were compared with anal condyloma acuminata that had no relationship with hemorrhoidectomy for microscopic comparison.

**Results:**

Of the 91 hemorroidectomies analyzed, eight had findings suggestive of viral cytopathic effects, with the presence of irregular acanthosis in 63%, koilocytes in 50% and other indirect viral cytopathic effects, such as hyperkeratosis (38%), parakeratosis (25% ) and papillomatosis (13%).

**Conclusion:**

This study was unable to conclude that there is an association between these two pathologic entities.

## Abstract (pt)

**Racional:**

Muitas pesquisas foram direcionados ao estudo da infecção do papilomavírus humano em região anal após a constatação de que é fator de risco para lesões precursoras e câncer anal.

**Objetivo:**

Estudar a associação existente entre achados histológicos sugestivos de lesões pelo vírus em espécimes de hemorroidectomias.

**Métodos:**

Foi realizado estudo de prevalência baseado em análise de laudos histopatológicos de hemorroidectomias com microscopia relatada para encontrar efeitos citopatológicos virais. Esses achados foram comparados aos laudos de condiloma acuminado anais que não possuíam relação com peças de hemorroidectomias para comparação de microscopia.

**Resultados:**

Dos 91 laudos de hemorroidectomias analisados, oito apresentaram achados sugestivos de efeitos citopatológicos virais, com presença de acantose irregular em 63%, coilócitos em 50% e os demais mostravam efeitos citopatológicos virais indiretos, como a hiperceratose (38%), paraqueratose (25%) e papipomatose (13%).

**Conclusão:**

Este estudo não pôde concluir ou inferir que exista associação entre essas duas entidades patológicas.

## INTRODUCTION

The genital infection by human papilomavirus (HPV) is the most frequent sexually transmitted disease in men and women. Nowadays, there are over 200 identified different subtypes, among which more than 100 are completely genetically sequenced, and more than 120 with partial sequencing. About 45 subtypes infect the epithelium of the male and female anogenital tract. Among these, 13 and 18 are considered of high oncogenic risk, mainly the 16 and 18 types, associated to the anogenital cancer and of the aerodigestive tract^ 2^^,^ 3 ^,^ 8 ^,^ 10_._

The anal HPV predominates in some groups, such as in patients carrying idiopathic anal itching, with antecedents of clinical form of anal HPV treatment, with antecedents of genital HPV treatment, who look for the ambulatories of sexually transmitted diseases, HIV positives, prostitutes and with risky behavior for sexually transmitted diseases. However, this disease also affects people who do not belong to these groups, and may be acquired by other forms beyond the sexual relationship^2^^,^ 6 ^,^ 7 ^,^ 8 ^,^ 10. 

The anal infection by HPV was now established as a risk factor for precursor anal injuries and anal cancer. Only a few investigators examined the prevalence and incidence of this anal infection, and the studies have been limited to the populations infected by HIV and immune depressed^2^^,^ 8 ^,^ 9 ^,^ 11. 

To the histopathology, the injury by HPV reveals koilocytosis. Initially, described by Leopoldo Koss^4^ in 1956, it consists of cells with pyknotic nuclei, surrounded by extensive clear halos with volume usually greater than the cytoplasm, seen in blades with optic microscopy. The koilocytosis constitutes pathognomonic signal of infection by HPV, serving as a basis for the studies of molecular biology^13^. 

It is also known that the HPV can show itself as a disease associated to a hemorrhoidal injury. In this context, the histopathology is very important due to the possibility of finding out that the injuries clinically diagnosed as simple hemorrhoidal processes, may be associated to several diseases, not only related to the HPV, but also parasitosis, mycoses, specific an nonspecific inflammatory processes, sexually transmitted diseases or carcinomas^1^. 

Among the anal diseases concomitant to the hemorrhoidal injuries, to the proctologic examination, it was found the presence of condyloma acuminatum as the fifth most frequent, being less prevalent than the anal fissure, hypertrophy of anal papillae or papillitis, anal fistulas and sphincter hypotony with partial anal incontinence. The finding of the presence of HPV in hemorrhoids was much higher even than the simultaneous cases of anal tumors^1^.

The aim of this study was to study the association among the histological findings suggestive of injuries by the virus in specimens of hemorrhoidectomies.

## METHODS

This research was approved by the Ethics Research Committee of the Federal University of Amazonas in Manaus/AM - Brazil, with the CAAE number: 15573913.4.0000.5020. It is about the transversal study or the presence prevalence of anal HPV, and of concomitant hemorrhoids. The data were collected from reports of histopathology of proctologic operations performed between 2003 and 2013. The presence of microscopic findings suggestive of HPV characterized by koilocytosis, parakeratosis, pyknotic nuclei surrounded by extensive clear halos with volume usually greater than the cytoplasms in parts of hemorrhoidectomies.

The inclusion criteria were: patients who had histopathology specimens or surgical fragments of elective proctologic operations of hemorrhoidectomy or condyloma acuminata. The exclusion ones, were patients who did not match the above criteria, and carriers of neoplastic processes associated with hemorrhoids. 

The target population was composed by all the patients who had histopathology of surgical proctologic specimens in the period, with the purpose to make an epidemiologic survey of the correlation between the development of hemorrhoidal injuries and the presence of the HPV virus. The variables were tabulated and analyzed through graphics. The possible associations were verified by means of the statistical test, considering level of 5% significance. The tests used were the chi-square and the exact of Fisher.

## RESULTS

Initially, 190 histopathology reports of patients who match the inclusion criteria of the research were selected. The origin of the surgical pieces was of 81% from the Getúlio Vargas University Hospital, and the others from other SUS hospitals.

**FIGURE 1:**
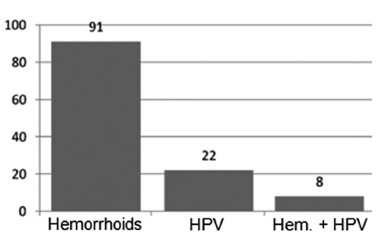
- Percentage of the diseases found in the anatomopathological reports

From the 190 selected reports, 153 were of hemorrhoidectomies and 37 of condyloma acuminata. However, 62 reports of hemorrhoidectomies were excluded from the research because they did not have microscopy data; 15 of condyloma acuminata were also excluded due to the absence of the microscopy description. Thus, 91 reports of hemorrhoidectomies, and 22 reports of condyloma acuminata were used, totaling 105 reports. Of the 91 reports of hemorrhoidectomies, eight had findings suggestive of viral cytopathic injuries in their microscopy, that is, 7.28% of the analyzed reports obeyed the association expected by the research (Figure 1).

**FIGURE 2:**
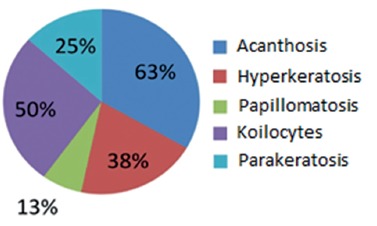
- Viral cytopathic findings suggestive of infection by HPV in pieces of hemorrhoidectomies.

On the eight reports of hemorrhoidectomies that had findings suggestive of viral cytopathic injuries (Figure 2), the more significant microscopic findings which indicated this injury were: acanthosis (63%); koilocytotic cells (50%); hyperkeratosis (38%); parakeratosis (25%); papillomatosis (13%); hypertrophic nuclei, hyperchromatics, loss of polarity and cell with the vacuolated cytoplasm (13%).

To measure the association between the two variables, the exact test of Fisher was used, which provided very low p, less than 2.2 x 10^-16^; by using the standard level of significance of 0.05, it was possible to conclude that the two variables have association.

On the 91 reports of hemorrhoidectomies, the most exuberant microscopy findings which characterized the histological diagnostic were: congested vessels (71%); dilated vessels (53%); ectasic vessels (36%); proliferation of fibrovascular tissue (35%); and thrombosed vessels (7%). 

On the 22 reports of condyloma acuminata of the comparison group, the most exuberant microscopy findings that characterized the viral cytopathic effect were: acanthosis (68%); hyperkeratosis (55%); papillomatosis (55%); cells with vacuolated cytoplasm (36%); koilocytes (32%); parakeratosis (27%); hypertrophic nuclei and perinuclear halo (23%); irregular acanthosis, hyperchromatic nuclei and cell binucleation (18%); loss of cell polarity (9%); vesicular nucleus (5%).

## DISCUSSION

For this research, the design of transversal study or of prevalence was used so that the degree of incidence and the association between these two entities could be analyzed: hemorrhoids and anal injuries by HPV.

According tom Cruz et al^1^, conclusive microscopy findings of viral injuries by HPV could be found in 0.4% of hemorrhoidectomy histopathology. This percentage of the study incidence performed in 2007, was much inferior to the percentage found in this research, which found cytopathic injuries suggestive of viral infection by HPV in 7.28%. 

On the eight reports that had findings suggestive of viral cytopathic injuries in their microscopy, 50% showed koilocytotic cells, which are classic cytomorphological findings of infection by HPV^8^^,^ 12; and beyond this finding, the description of the microscopy also related indirect cell injuries, also considered classics in the infection by HPV, such as the hyperkeratosis (38%), parakeratosis (25%); papillomatosis, hypertrophic nuclei, hyperchromatic found in 13% of the cases.

According to Toro et al^12^, on the microscopic analysis of HPV blades, koilocytes were found in 69.1% of the cases, parakeratosis in 33.3%, and nuclear hyperkeratosis in 28%. These results are in accordance with the data found in the analyzed pieces of hemorrhoidectomies, practically with the same percentage of the findings in microscopy in HPV blades.

On the 22 reports of the comparison group that had exclusive diagnostic of condyloma acuminata, the most significant microscopic findings that characterized the viral cytopathic effect are also in accordance with the study cited before^11^, which classifies the presence of koilocytes and/or diskeratocytes as classic cytomorphological findings of infection by HPV, and that this virus can also induce the appearance of indirect cell alterations like: binucleation, megalocytosis, chromatin cells slightly irregular, hyperkeratosis and parakeratosis, nuclear hyperchromasy and megalocytosis.

Thus, the most significant finding of cytopathic injury by HPV, which is considered as the presence of koilocytes in optic microscopy, was found in only 32% of the comparison group whose diagnostic was of condyloma acuminata, and cited in the microscopy of 50% of the hemorrhoidectomy reports that had no diagnostic of HPV.

Based on the result of these studies, it is noted that the histopathology may be a very important tool in the diagnostic of cases of viral injuries by HPV even before their clinical manifestation, from a careful assessment in optic microscopy of surgical pieces and tissues fragments removed from the anal region^5^^,^ 7 ^,^ 8 ^,^ 9 ^,^ 10 ^,^ 11.

Proportionally, the coexistence of hemorrhoids and microscopic findings suggestive of viral cytopathic injuries was noted in every 11.37 cases of hemorrhoids. Even if the measure of association calculated by the exact test of Fisher has provided very low p, it was not possible to ensure that there is any association between these entities, due to the several biases negatively influencing on the results. Among them, we may cite the lack of confirmation of the presence of HPV in the pieces of hemorrhoidectomy, once the microscopy analysis cannot singly be a diagnostic parameter for this disease and, therefore, cannot be deliberately included into the case group where the two diseases coexist. Another bias found was of selection, because the number of reports present in the comparison (reports of condyloma acuminata singly) was much smaller than the number of evaluated cases. This bias happened because the number of pathological examinations of hemorrhoidectomy is much higher in relation to the ones of condyloma acuminata. The bias of the observer was also found in the study because the reports were not analyzed nor emitted by the same pathologist, and not confirmed by other pathologists.

It is important to stress that the aim of this research was not to attest the HPV as a precursor of hemorrhoidal injuries, even because the method of epidemiological study chosen was the transversal or of prevalence, which is analytic, evaluating only the distribution of the frequency of a particular disease in relation to the time. This type of study allows only the analysis of the association between two events, being able to measure the frequency in which both may occur at the same time, and if this association may be significant; but it does not permit the etiologic assessment of the studied event.

The study of prevalence was chosen because it estimates, on a satisfactory way, the proportion of exposed ones to a particular disease, in the population; it is good to program public health policies with the purpose of an early diagnostic of several diseases. 

The main goal of this paper was to draw attention to the quantity of subclinical cases of HPV, that may no longer be early diagnosed due to the simple lack of more specialized histopathology that differentiates other anal injuries^5^^,^ 7 ^,^ 8. Attesting the casual relation between HPV and hemorrhoids was not the aim of this study.

## CONCLUSIONS

This study could not ensure or infer that there is any association among injuries by human papilomavirus in specimens of hemorrhoidectomies.
